# Dataset of sodium chloride sterile liquid in bottles for intravenous administration and fill level monitoring

**DOI:** 10.1016/j.dib.2020.106472

**Published:** 2020-10-31

**Authors:** Danilo Pau, Bipin P. Kumar, Prashant Namekar, Gauri Dhande, Luca Simonetta

**Affiliations:** a System Research and Applications, STMicroelectronics, Agrate Brianza, Italy; b Sesovera Tech, Bengaluru, India

**Keywords:** Fill level of bottles, Sodium chloride liquid, Saline solution, Visual monitoring

## Abstract

We propose a dataset to investigate the relationship between the fill level of bottles and tiny machine learning algorithms. Tiny machine learning is represented by any Artificial Intelligence algorithm (spanning from conventional decision tree classifiers to artificial neural networks) that can be deployed into a resource constrained micro controller unit (MCU). The data presented has been originally collected for a joint research project by STMicroelectronics and Sesovera.ai. This article describes the recorded image data of bottles with 4 levels of filling. The bottles contain sodium chloride sterile liquid for intravenous administration. One subject of investigation using this dataset could be the classification of the liquid fill level, for example, to ease continuous human visual monitoring which may represent an onerous time-consuming task. Automating the task can help to increase the human work productivity thus saving time. Under normal circumstances, human visual monitoring of the saline level in the bottle is required from time to time. When the saline liquid in the bottle is fully consumed, and the bottle is not replaced or the infusion process stopped immediately, the difference between the patient's blood pressure and the empty saline bottle could cause an outward rush of blood into the saline.

## Specifications Table

**Table:** 

Subject area	Computer Science
More specific subject area	Computer Vision and Pattern Recognition
Type of data	Image dataset (.jpg), Secondary data: images labels (.txt), jupyter notebook (.ipynb)
How data were acquired	All images were captured from a Realme X2 mobile phone. The camera specifications are 64 MP + 8 MP + 2 MP + 2 MP (MegaPixels) Quad Primary Cameras. No flash was fired while capturing images.
Data format	Images dataset: raw data (.jpg) organized in folders named by filling level (empty, 50%, 80%, 100%) Secondary data: ipynb file (jupyter notebook), .txt (images labels)
Parameters for data collection	The standard bottles were photographed from different directions and places with various backgrounds. The bottles were placed at random distances and angles from the camera in every image. Images were captured having adequate amounts of light. Efforts were made to get bubble free and straight levelled saline water in the bottle. The liquid in the bottle was kept at different levels of fill percentage (empty, 50%, 80%, 100%).
Description of data collection	Multiple images were acquired by placing a limited number of standard bottles upside down with different perspectives and backgrounds, no flash was fired during image capturing process. Furthermore, data were augmented using python script which is part of the dataset (released as jupyter notebook) and a txt file used to augment and resize data. As part of the augmentation process, the image data set can be pre-processed, optionally, with a negative filter, resized to 64 × 64 pixel and augmented with different procedures that concerned zoom, rotation, shear, horizontal flip, width and height shift.
Experimental factors	Furthermore, to ensure its practical usability, the dataset has been tested with an exemplary convolutional neural network.
Data source location	Institution: Sesovera ai Country: India Latitude and Longitude for collected data: 12.972442, 77.580643
Data accessibility	Name1: SESOVERA-ST-saline-bottle-fill-level-monitoring:empty_50%fill Name2: SESOVERA-ST-saline-bottle-fill-level-monitoring:80%fill_100%fill Direct link to data1: http://dx.doi.org/10.17632/n8k2zfr6xm.1 Direct link to data2: http://dx.doi.org/10.17632/9mcj3rvvxb.1

## Value of the Data

• The dataset described and provided with this paper supplies valuable information to investigate the opportunity to enable classification with resource constrained micro controllers thus easing human attention to that onerous task. The example considered is of detecting the quantity of saline (sodium chloride) in a bottle used for intravenous administration for medical purposes. Automating the visual monitoring of the bottle in its environment helps nurses and other human operators to increase their work efficiency, reduces time and saves lives. Under traditional circumstances, continuous visual monitoring of the saline level in the bottle is required to be performed by humans.
• When the saline in the bottle is fully consumed, and the bottle is not replaced or the process stopped immediately, the difference between the patient's blood pressure and the empty saline bottle could cause an outward rush of blood into the saline.
• The dataset can be used to investigate the capability of a resource constrained micro controller unit to deploy the result of machine learning, pattern matching both classification and object detection. The data are also suitable for different pattern recognition tasks such as per pixel classification and bottle vs background segmentation.

## 1 Data Description

The image data, for each bottle fill level, provides different perspectives, illumination conditions, focus on the bottle, background. These are useful to challenge the visual evidence of the saline liquid level inside the bottle.

The dataset proposed consists in an archive of 4217 images. As shown in Table 1, the file named "saline_bottle_original_size.zip" contains a total of four folders as it shown inFig. 2, namely:

1 "sal_data_empty": contains empty saline bottle images.
2 "sal_data_50": contains saline bottle images with 50 percent filled.
3 "sal_data_80": contains saline bottle images with 80 percent filled.
4 "sal_data_100": contains saline bottle images with 100 percent filled.

**Table 1:** Number of images composing the dataset.

Class ID	Class descriptionFill level	Number of original images at 3456 × 3456 pixel resolution	Number of images at 64 × 64 pixel resolution after data augmentation
1	Empty bottles	1054	9936
2	50%	1052	9927
3	80%	1054	9999
4	100%	1057	9982

The provided .ipynb file in the saline directory is useful for building the software pipeline that operates on the dataset. This pipeline involves the creation of txt file used to manipulate images, build the structures suitable to host the resized dataset and provide functions aimed to augment the dataset. Table 1 shows the size of the dataset before and after the augmentation and resizing procedure from 3456 × 3456 pixel resolution down to 64 × 64.

**Fig. 1:**
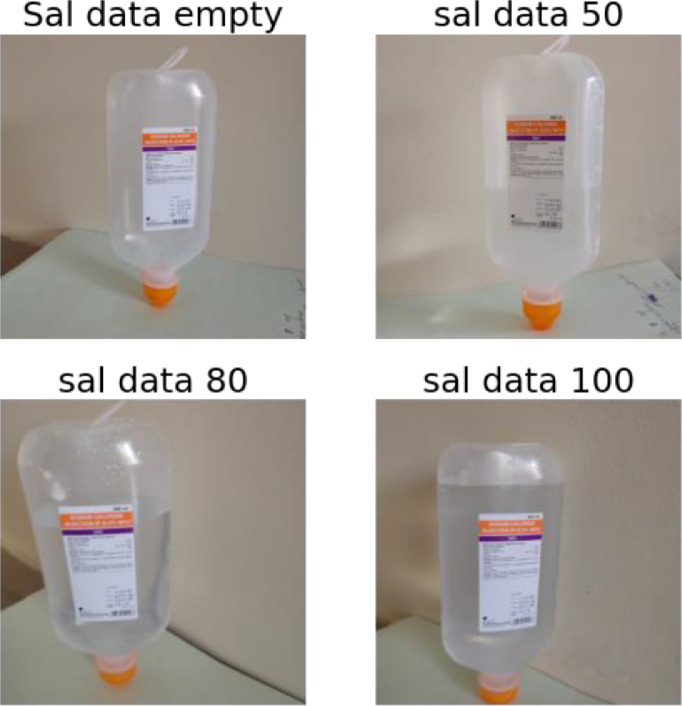
Exemple of images data capture for each class ID and fill level.

**Fig. 2:**
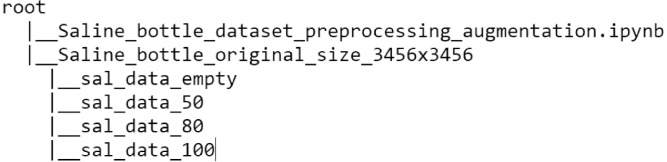
Contents of saline_bottle_original_size.zip.

### 1.1 Type of bottles

As example, a typical saline (sodium chloride NS) 500 ml water bottle was acquired on the market. It was used for obtaining samples and creating the dataset. The body of the bottle was made of soft transparent plastic, which enabled us to acceptably capture the saline water level. The bottle had a milliliter (ml) scale printed on a side, which was used to measure and calibrate the level of saline water accurately. Following there are the levels of saline water, starting from zero (bottle upside down):

1 Empty: 0 ml of saline water.
2 50 percent full: 250 ml of saline water.
3 80 percent full: 400 ml of saline water.
4 100 percent full: 500 ml of saline water.

## 2 Experimental Design, Materials and Methods

### 2.1 Acquisition procedure

**Fig. 3:**
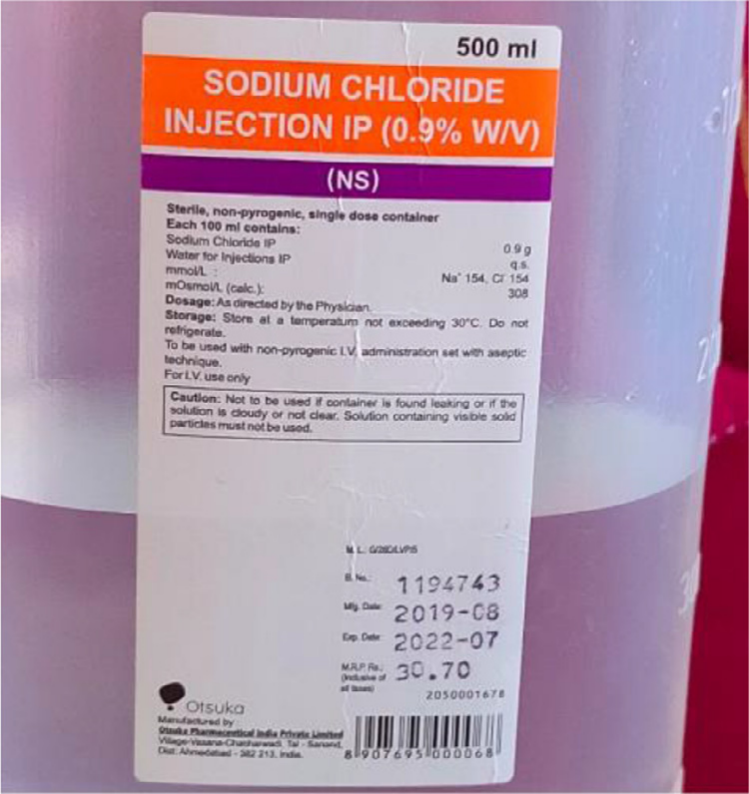
Exemplary view of the saline bottle.

**Fig. 4:**
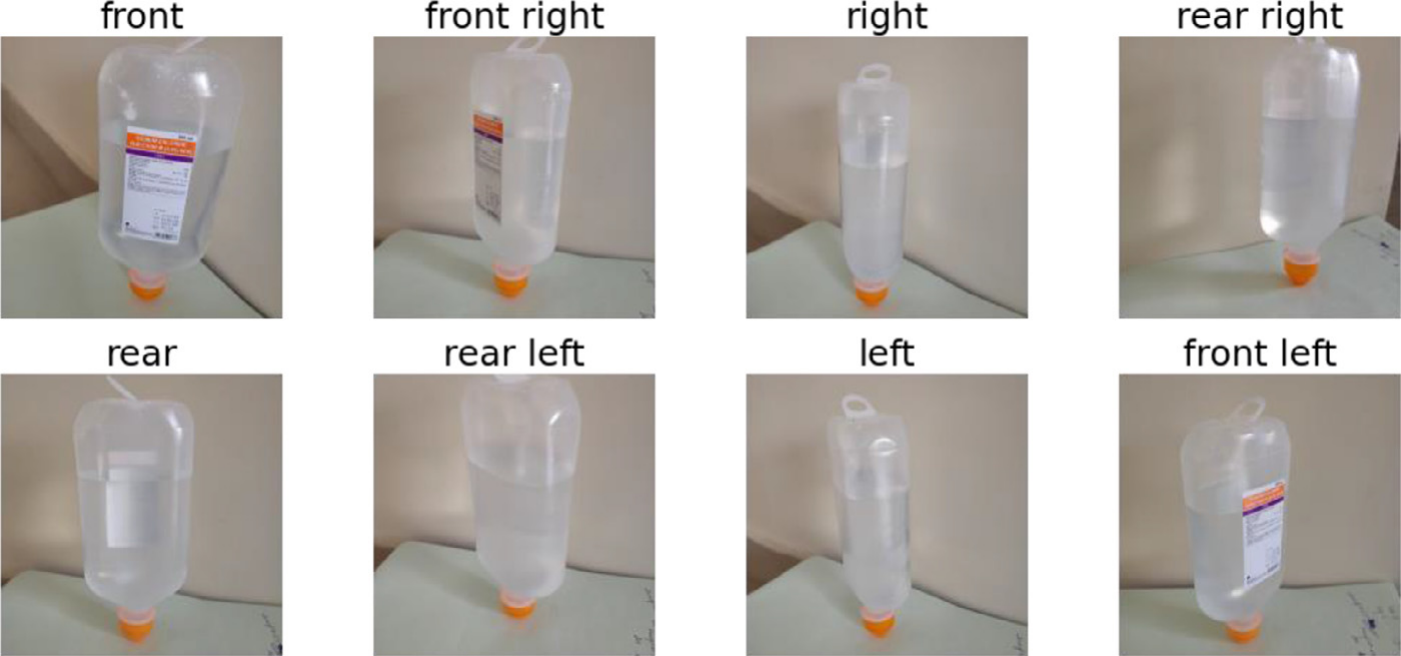
Image examples of standard bottles with white wall as backgrounds at random distances and angles.

All images are in jpg (Joint Photographic Experts Group) format captured from a Realme X2 mobile phone. The camera specifications are 64 MP + 8 MP + 2 MP + 2 MP Quad Primary Cameras. No flash was fired while capturing images. The images were acquired by placing the bottle upside down. Multiple Images were captured using a limited number of standard bottles at different places and vantage points with various backgrounds. The standard bottle is characterized by a label placed on it that shows the specifications of the saline solution as it is shown in Fig. 3. For example, the same bottle was positioned at random distances and perspectives, then captured by the camera as shown in Fig. 4. Efforts were made to get bubble free and straight levelled saline water in the bottles. Images were captured having adequate amounts of light to make the level line appear clearly.

**Fig. 5:**
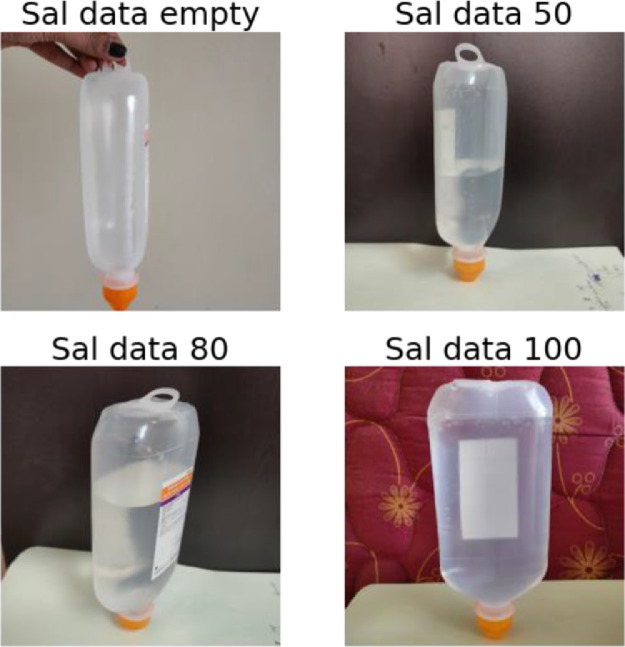
Image examples of standard bottles with different type of backgrounds at random distances and angles.

Different types of images were captured for example holding the bottle with the hand or lying on a notepad with a white wall, red texture or wooden panel as background as shown in Fig. 5.

### 2.2 Data pre-processing procedures

**Fig. 6:**
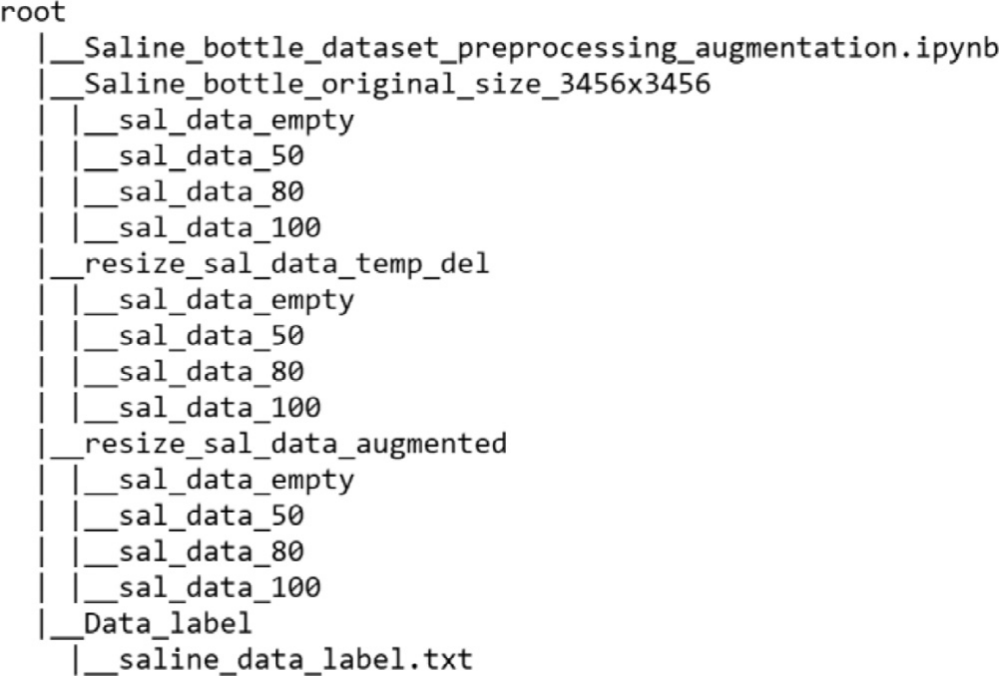
Directories tree generated with .ipynb provided with the dataset.

The preprocessing allows to manipulate the dataset and carry out procedures aimed to data augmentation. In the .ipynb file (available as part of the dataset) executable steps are enclosed that allow, having set the path of the dataset, to create a dictionary with list of images labels by organizing them in various directories depending on filling level of the bottles. Fig. 6 shows the complete directory tree generated from the .ipynb provided with the dataset. The images are manipulated with the support of saline_data_label txt file (generated in Data_label folder) which keeps track of them and allows labeling by directory. For example, images residing in sal_data_50 folder will have a corresponding label that marks them as bottles 50 percent full. The proposed workflow after creating folders, label the images contained in them with associated ID for each fill level: sal_data_100, {0}; sal_data_50, {1}; sal_data_80, {2}; sal_data_empty, {3}. After this operation is done, the workflow continues with the creation of two structures, one vector for the representation of the dataset images, one array contains the images ID labels. The pipeline proceeds with the optional application of filters on images, it also computes the negatives, because it allows to highlight the thin lines representing the bottles filling level. Finally, the augmentation procedure by increasing by 10x the images count, applies slight changes of the image orientation, as well as zoom and shift. The pre-processing procedures were carried out using a) Python Image Library PIL ‘7.0.0’ library [1] to manipulate images and using associated facilities, b) keras ‘2.4.3’ and c) numpy ‘1.18.5’ libraries to support reshape and augmentation procedures.

#### 2.2.1 Preprocessing procedure

**Fig. 7:**
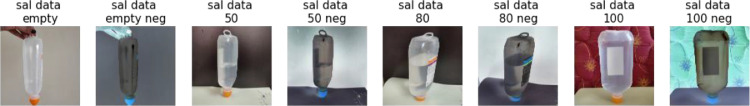
Example of negative transformation applied on images of bottle saline dataset shown in Fig. 4.

The images have been preprocessed with a negative filter by using PIL. This visualization had been done on purpose to emphasize the transparency of saline solution's filling level in a different way from the original color as shown in Fig. 7. In the python script provided, there is a procedure to produce the negative of any of the images, save them in a file and with the possibility to convert the images in different color space, for example, to YCrCb.

#### 2.2.2 Resize procedure

PIL is also used to resize images. The images were resized to 64 × 64 pixels as default, but it also allows to target a different resolution. As a result, the size of each image footprint reduced from 3.1 MB to approximately 1.3 Kbytes. The resized format remains as original, default .jpg but the python code provided is also meant to allow conversion to other color space. All these images can be saved in a temporary folder (resize_sal_data_temp_del as it is shown in directories tree in Fig. 6) with their respective original sub folders and names.

#### 2.2.3 Augmentation procedure

**Fig. 8:**
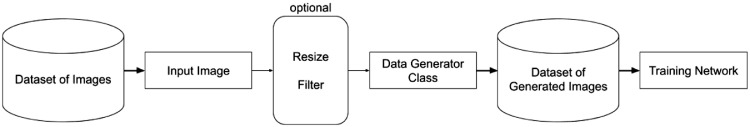
Data preprocessing pipeline before network training.

Data Augmentation encompasses a suite of techniques that enhance the size and quality of training datasets such that more robust and accurate Deep Learning models can be built using them [2]. The augmentation of images is performed a-head of training the neural network as it shown in Fig. 8. The procedure acts on some parameters such as rotation range angle of 40°, width shift range of 0.2, height shift range of 0.2, zoom range of 0.2 and horizontal flip allowed. It should be noted that the class returns only the augmented images and not the original images. As the number of samples in the dataset increased, it is expected that the model can achieve better accuracy under more general working conditions.

**Fig. 9:**
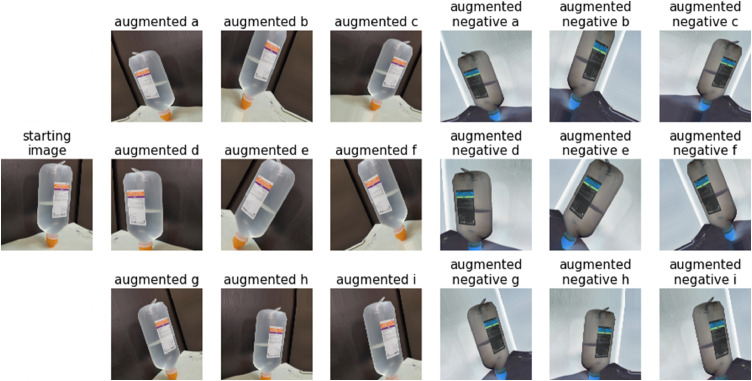
Examples of augmentation on a starting image with fill level 50%; augmented a,b,c,d,e,f,g,h,i are the augmented images; augmented negative a,b,c,d,e,f,g,h,i are the augmented images with negative pre-processing and no image resize.

Keras (v 2.4.3) deep learning library provides a class ImageDataGenerator, which we used for generation of more images and configure random transformations and normalization operations [3] as shown in Fig. 9.

Python code released with dataset can perform different actions depends on the purposes of the user:

• Importing libraries such as Keras and PIL, useful to execute the pipeline aimed to resize and augment dataset.
• Possibility of using YCrCb or RGB color space and negative of images.
• Possibility of using different image resolutions.
• Possibility to achieve augmentation by creating ImageDataGenerator's object class and setting the required parameters such as zoom, rotation, shear, horizontal flip, width and height shift.
• Set image path and path were images need to be stored.

### 2.3 Exemplary performances achieved by a neural network

**Fig. 10:**
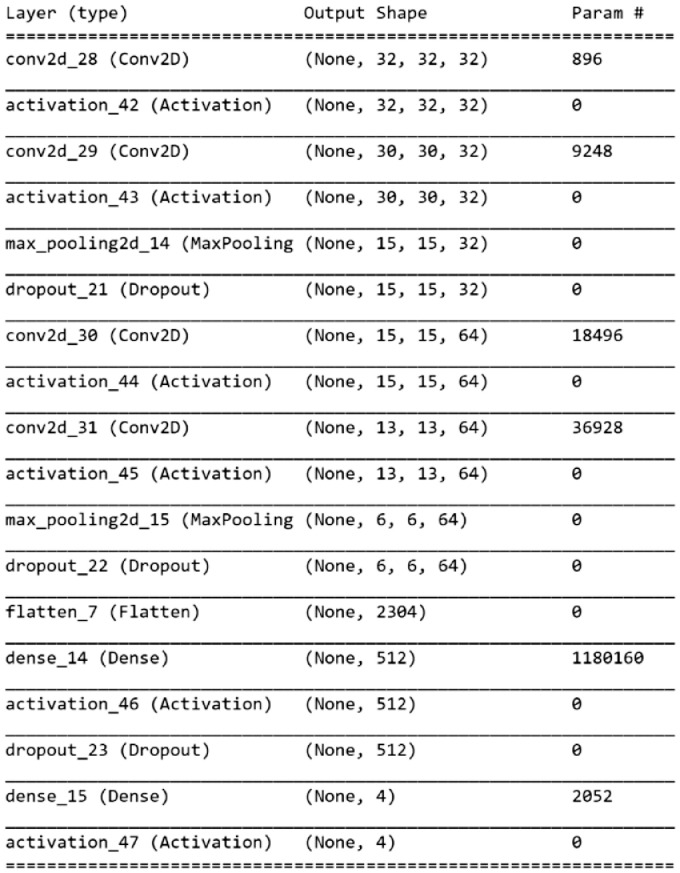
Exemplary baseline network model summary.

We trained a convolutional neural networks using this dataset to demonstrate an example of performances that can be achieved. Accuracy and loss achieved are also reported, although describing them in detail is not in the scope of this paper. Table 2 shows the accuracy and loss values resulting from an example baseline model trained without augmentation using a network structure consisting of 4 convolutional 2D layers featuring kernel size 3 × 3 followed by ReLu activation function. Fig. 10 shows the Keras model summary with the number of parameters, the use of 2D max pooling with 2 × 2 pool size and dropout after ReLu non-linearity. Finally, the latest 2D Max pooling layer which calculates the maximum value for each patch in feature map is followed by a flattening, dense and Softmax layer to classify the provided input training images into 4 fill level classes. The last max pooling stage is also useful for summarizing the characteristic detected by the convolutional layer and simplifying the task of the classifier. The results of the executions show that the network classified the images accurately enough; however it is certainly subject to improvements by the AI community so as to increase the accuracy and decrease reported loss.

**Table 2:** Loss and accuracy measurements of the exemplary baseline model obtained with 3 random executions.

Exemplary Model	Execution	Loss	Accuracy	Epochs
	exec 1	0.236	0.938	131
	exec 2	0.218	0.940	124
	exec 3	0.281	0.938	106

## Declaration of Competing Interest

The authors declare that they have no known competing financial interests or personal relationships which have, or could be perceived to have, influenced the work reported in this article.
